# The variation differences of cultivated land ecological security between flatland and mountainous areas based on LUCC

**DOI:** 10.1371/journal.pone.0220747

**Published:** 2019-08-01

**Authors:** Li Wu, Binggeng Xie

**Affiliations:** 1 Department of resources and environmental sciences, Hunan Normal University, Changsha, China; 2 Department of geography and land engineering, Yuxi Normal University, Yuxi, China; Institute for Advanced Sustainability Studies, GERMANY

## Abstract

The purpose of this study was to explore the correlations between land use/cover change and cultivated land ecological security in flatland and mountainous areas. Firstly, the spatiotemporal variation characteristics of land use/cover change are described in conjunction with ArcGIS10.5 software based on remote sensing images of 2005 and 2015. Then, by establishing a pressure-support framework as an assessment indicator system and developing an improved BP neural network model via a genetic algorithm with the help of MATLAB2016a, the spatiotemporal dynamic changes of cultivated land ecological security in Yuxi City from 2005 to 2015 are evaluated. The results showed that the transformation of farmland area accounted for a large proportion of increased constructive land and land use/cover spatial variations were significantly different among counties, which manifested the changes in farmland and the construction land in flatland areas but also facilitated a mutual transformation of forest and grass in mountainous areas. Moreover, ecological security status presented a clear difference among counties due to their different land use/cover changes. The ecological security state of the flatland expressed a higher ecological pressure and lower ecological support, so the security grade was IV. Otherwise, the ecological security was superior and the security grade was level II or I in the mountainous areas. Thus, protection strategies for ecological security should be differentiated in the flatland areas and mountainous areas due to their different ecological security status brought by land use/cover change.

## Introduction

As essential components to balance the lives on Earth, cultivated land plays important roles in food production, ecological services, social stability, national security and landscape aesthetics values[1]. However, despite their benefits, farmlands are also very fragile ecosystems that are threatened by overexploitation and urban land expansion. Early in 1998, Brad Allenby presented that the relationship between environmental security and industrial development was profound[2]. The economic development strategy[3] and competitive land use[4] limited land ecological protections. The land use/cover change(LUCC) has significant impacts on ecological environments[5, 6], as fertile farmlands were increasingly swallowed by built-up land and growing contamination; these contaminants included the abuse of pesticides, artificial fertilizers, agricultural films[7], and heavy metal pollution, which led to the declination, degradation and desertification of the cultivated land. Alterations of the cultivated land quality and quantity by human activities, including transforming the land use/cover, can potentially degrade ecological security (ES) and undermine the ecological carrying capacity in the future [8]. A secure ecosystem is obviously the foundation of a sustainable cultivated land production capacity. Thus, the ecological security and sustainable development of the cultivated land should be increasingly focused on and become a current and future research priority. Assessments and researches on the past changes and current status of cultivated land ecological security can provide more targeted references for utilization and management in the future.

In 1977, Constance Holden appealed that the redefining of national security should be related to ecology and change the traditional equation with military might[9]. With rapid economic development, human living space has become unprecedentedly challenged, which aroused increasing and urgent concerns towards ecology security from both local and worldwide scholars. Based on the definition of land ecological security [10], the ecological security of cultivated land as an important component of a land ecosystem can also be defined by natural, economic and social security with a healthy and balanced structure and function within the spatial and temporal ranges. If the three aspects of a cultivated land system are in good condition and the resources are not overexploited or degraded by human activities or other natural disasters in this region, then the cultivated land system in this region is ecologically secure. According to the previous researches and the theory of ecosystem balance, the concept of cultivated land ecological security can also be defined as the ecosystem balance that is divided into a cultivated land eco-pressure subsystem and eco-support subsystem in this study. The assessment index systems have been proposed for the various perspectives of nature, economy and society (NEC) [11]; pressure, status and response (PSR) [12, 13]; or improved, modified, and combined frameworks based on the two models, which included the DPSER model (driver-pressure-state-exposure-response)[14], the DPSIR (Driver-Pressure-State-Impact-Response) framework[15], and the PSIR (Pressure-State-Impact-Response) model[16]. The two subsystems that are composed of the cultivated land eco-system, named the eco-pressure subsystem and eco-support subsystem, can graphically describe the status of eco-security more. Thus, the assessment index system that will be set up from these two subsystems can be considered as a PS framework.

Methods that applied to be calculated the security index and graded security level varied from comprehensive evaluations[10] to landscape ecology [17] and ecological models based on various mathematical models and algorithms, such as hierarchical patch dynamics[18], ecological footprint theory[19, 20], the cellular automata model[21, 22], GDHAS combined GTT and AHP[18], catastrophe theory[11, 16], the Least-Cost Path (LCP) model[23], minimum accumulative resistance (MCR) [24, 25] and the logistic regression model[26]. Moreover, the back-propagation (BP) neural network, which can approximate any nonlinear function and is widely applied in predictions, optimizations, assessments and classifications in China, has been used less in ecological security evaluations[27]. A cultivated land ecological system is a complex multivariable nonlinear dynamic system, that has posed many limitations on traditional methods for assessing and analyzing, and the BP neural network has strong nonlinear approximation ability and the capabilities to handle unclear, disordered and complex information[28]. Its characteristics are appropriate for overcoming the shortcomings of traditional approaches, thereby reaching a higher accuracy in a short time. At present, many existing studies have verified that the BP neural network was feasible for evaluating and predicting the ecological security and equality of water[29] and land resources[30]. However, the traditional BP neural network has many defaults, such as a slow learning convergence, falling into local extremum easily and overfitting in algorithms, but an algorithm optimization can balance the capabilities of exploration and development[31]. The previous studies have applied a BP neural network improved with a genetic algorithm (GA) in predictions and assessments in many fields. When compared with the traditional BP neural network, the improved GA-BP neural network not only has a better stability and global searching ability but also has the capacities for automatic acquisition, accumulating spatial knowledge and adaptively controlling the search process. These enhancements greatly improved the network performance. Although it has many advantages, the GA-BP neural network has rarely been applied in cultivated land eco-security evaluation with limited information.

The cultivated land is an open land use system that not only has certain interactions with human activities and society economic development, but also interacts with variations of other land use/cover types in different terrains. Two typical terrains are flatland and mountain land formed mountainous areas. Because their differences are rooted in different human activities, flatland and mountain land vary differently in LUCC through various kinds of human development stages[32]. As previously mentioned, LUCC plays an important role in changing the ecological environment via human activities. Therefore, different dynamic variations in the flatland and mountain land can bring different changes in the cultivated land ecological security. Analyzing in different dynamic variations of LUCC and cultivated land ecological security not only can reveal the interrelationships between LUCC and land ecological security but also can provide some references for differentiation development measures to development of flatland and mountainous areas. This study focuses on different variations of cultivated land ecological security dynamics evolutions and their correlation with LUCC between the flatland and mountain land, and the methodology is as follows: First, according to the definition of cultivated land ecological security, a pressure-support (PS) framework based on PSR model was originally proposed as an assessment indicator system. Then, based on the remote sensing image as the data source, the results of the transfer matrix of land use/cover changes from 2005 to 2015 were obtained to analyze variation characteristics. Moreover, an improved BP neural network model based on an adaptive genetic algorithm was developed to calculate the cultivated land ecological pressure index and support index, which can calculate the ecological security index and evaluate the security grade, respectively. Last, the causes of ecological security variations and correlations between ecological security changes and LUCC from 2005 to 2015 and different variations between flatland and mountainous areas were all analyzed.

The aims of this paper are: (1) to apply a PS assessment index system to evaluate the cultivated land ecological security based on the Pressure-Support (PS) frame work; (2) to reveal the temporal-spatial characteristics of LUCC from 2005 to 2015 in the flatland areas and mountainous areas of Yuxi City; (3) to compute the ecological pressure index and ecological support index based on a BP neural network improved with a genetic algorithm; and (4) to analyze the temporal-spatial dynamic variations of cultivated land ecological security and their correlations with LUCC over the last 10 years in Yuxi City and every county.

## Materials and methods

### Study area and data sources

#### Regional overview

**Fig 1:**
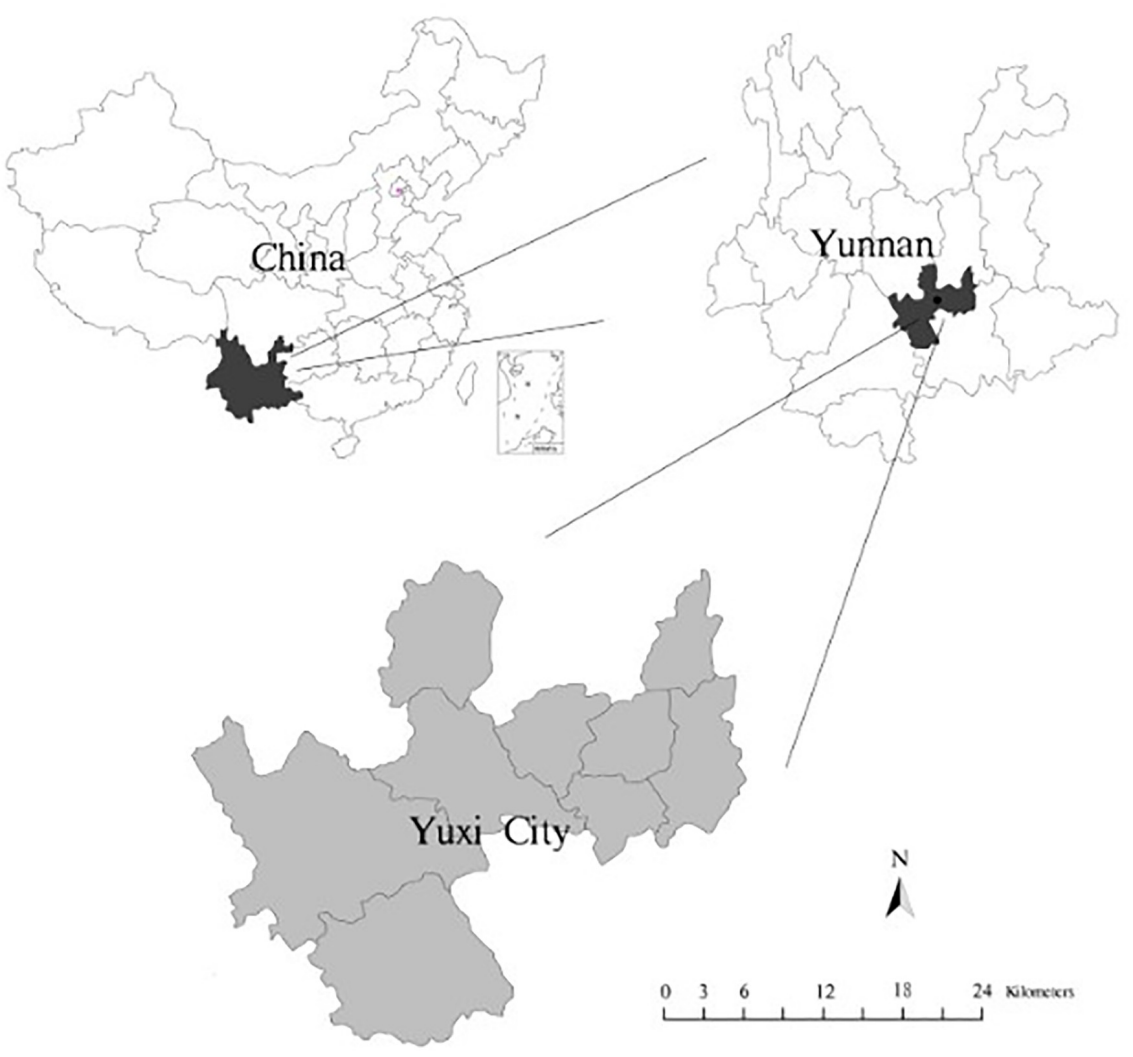
Location of the study area. The location of Yuxi City in Yunnan Province and China. The image was obtained by using ArcGIS 10.5.

Yuxi City is located in the center of Yunnan Province (Fig 1) and between 101°16′E~103°09′E and 23°19′N~24°53′N, and is high in the northwest, and low in the southeast. As a typical mountainous area, the topography is complicated, and the mountains, canyons, plateaus and basins are staggered, which also bring complexity and vulnerability to the land utilization. Yuxi City has rich natural resources, including mineral resources, forest resources and water resources. The mountain stereo climate, which is a moderate climate that is neither severely cold in winter, nor overly hot in summer and unique ethnic customs attracted increasing numbers of local and international visitors to study or relax in the area, which facilitated the development of the local society and economy.

By 2015, the urbanization level of Yuxi City reached 46.9%, which is an increase of more than 20%, and the GDP per capita, quadrupled over 10 years. However, the fragile land resources and ecological environment of the mountainous area also face severe challenges over increasing human impacts, especially the cultivated lands that have widely been exhausted and therefore have become increasingly precious land resources. To protect an area’s ecological security is to improve its social and economic sustainability, so there is an urgent need for cultivated land ecological security evaluations to guide decision-makers on effective land use.

#### Data sources and processing

Our land use/cover change analysis takes 2005 and 2015 Landsat-TM images as data sources as well as a classified statistical table of land use status, statistical year book (from 2005 to 2015), a land use planning map and relevant research data as auxiliary information in this paper. The regional character of Yuxi City combined with the clarity degree of the remote sensing images, visual interpretation and field survey data are adopted to set up the interpretation mark. Land use/cover types in Yuxi City are classified as farmland, forest, grass, water, and construction land. After classification and interpretation, the land use data in 2005 and 2015 are compared with the data from the Natural Resources Bureau of Yuxi City. The results showed a higher interpretation accuracy and reliable classification. In ArcGIS10.5, two periods of land use/cover data are obtained, and an analysis and calculation of the classified statistics and spatial overlay are all prepared.

The ASTER GDEMV2 global digital elevation database is chosen as DEM data, which comes from the Geospatial Data Cloud of Computer Network Information Center (http://www.gscloud.cn). First, the data are preprocessed with the mosaic and cut tools of ArcGIS10.5, then, the slope data and topographic relief are acquired by applying the surface analysis tool. Afterwards, the definition projection, reclassifying, raster to polygon and cut tools are applied to obtain the different slopes and topographic reliefs of different counties in Yuxi City.

The information on human activities, society, economics and the nature factor index, including population density, multi crop index, population support per unit grain crops sown area, GDP growth speed, rural erosion rate, pesticide load per unit cultivated area, fertilizer load per unit cultivated area, mulching film load per unit cultivated area, forest coverage rate, green investment accounts for GDP, effective irrigation area rate, soil erosion control rate, comprehensive utilization ratio of industrial solid wastes, annual precipitation and per unit area grain yield of cultivated land are mainly derived from the Yuxi Statistical Year Book (2005–2015) and the corresponding statistical bulletin.

### Methods

#### Establishment of evaluation index system

**Fig 2:**
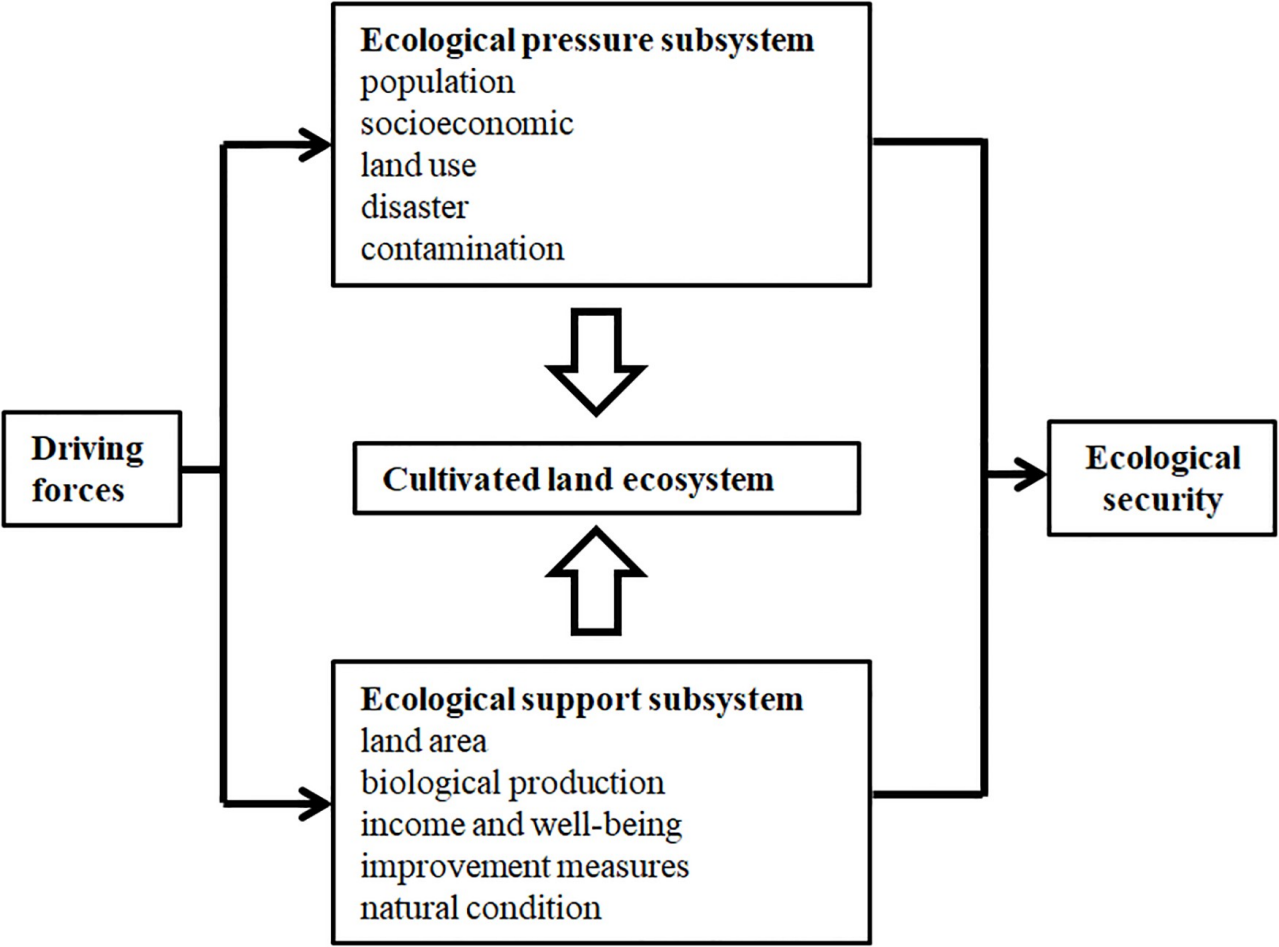
Pressure-support (PS) framework for cultivated land ecological security. Broad arrow pointing down refers to the pressures on the cultivated land ecosystem and broad arrow pointing up refers to the support to the cultivated land ecosystem.

**(1) Determination of the evaluation framework**: As a sustainable evaluation index system, “Driving-State-Response” (DSR), was proposed by the Commission on Sustainable Development(CSD) in 1996 and was then developed into the “Pressure-State-Response” model (PSR) by OECD and the United Nations UNEP[33]. Due to its advantages of integrity and flexibility, the PSR model became the most widely applied framework for establishing evaluation index systems in ecology and other fields and providing theoretical proofs for sustainable development [18]. It can represent the pressure of human activities on the environment and the environmental state after stress[34]. Ecological balance should be defined as the balance of import and export[35]. So, based on the PSR framework, an improved indicator system named PS model is proposed in this study as follows (Fig 2). A cultivated land ecosystem can be divided into two subsystems, an ecological pressure system and an ecological support system (PS); if the impacts of the support system can weaken or at least balance the harmful influences from the pressure system, then the cultivated land system can be considered secure and vice versa.

**(2) Selection of ecological evaluation indexes**: Based on PS framework model, the features of economy, society and ecological environment in Yuxi City are analyzed. Considering the comparability and accessibility of evaluation indicators, 18 indicators are chosen to establish the assessment system from two subsystems and six dimensions. According to the previous analysis, the cultivated land ecological pressures of Yuxi City are mainly from an increase in population demand, development in society and economy, natural disaster and environmental pollution. Therefore, the pressure subsystem is composed of socioeconomic pressures, disaster pressures and contamination pressures. Nine representative eco-pressures indicators are chosen to reflect the pressures on the cultivated land ecosystem. The support subsystem consists of state supports, management and protective supports, and conditional supports. From land type structure, investment in environmental protection and natural conditions, nine eco-support indicators are chosen to measure the supporting capacity of the cultivated land ecological system (Table 1).

**Table 1:** Eco-security evaluation indicators system of cultivated land.

Dimensions	Factors	Indicators	Weight
**Ecological Pressure subsystem**	Socioeconomic pressure	Population density (people/km^2^)	0.2541
		Multi crop index (%)	0.0721
		Population support per unit grain crops sown area (people/hm^2^)	0.1793
		GDP growth speed (%)	0.0105
	Disaster pressure	Rural erosion rate (%)	0.0523
		Land reclamation rate (%)	0.1114
	Pollution pressure	Pesticide load per unit cultivated area (kg/hm^2^)	0.0671
		Fertilizer load per unit cultivated area (kg/hm^2^)	0.1270
		Mulching film load per unit cultivated area (kg/hm^2^)	0.1262
**Ecological Support subsystem**	State support	Forest coverage rate (%)	0.0712
		Ecological land index (%)	0.0882
		Land use type diversity index	0.0700
	Management and protection support	Green investment accounts for GDP (%)	0.4251
		Effective irrigation area rate (%)	0.0722
		Soil erosion control rate (%)	0.0974
		Comprehensive utilization ratio of industrial solid wastes (%)	0.0775
	Condition support	Annual precipitation (mm)	0.0421
		Per unit area grain yield of cultivated land (kg/hm^2^)	0.0563

Notes: GDP refers to gross domestic product and the abbreviations are also used for the following contents.

(3) Calculation of partial indexes

Most of the indicators can be calculated with conventional formulas, but some that should be interpreted for their specific function and computed are described here.

Ecological land index: Among the land use types, forest, water body, grassland and other lands called ecological land[27] and played very important roles in ecological security, so their proportion within the whole structure can indicate the grade of ecological security. The coefficient of the land use structure, which is defined as the proportion of ecological land, can be calculated by the following formula:

$$M_{i}=\frac{\sum E_{l}(forest,waterbody\ldots )}{S_{t}}$$ (1)

Where *M*_*i*_ is the ecological land ratio of the *i*th area, *E*_*l*_ is the ecological land area, and *S*_*t*_ is the total land area.

Land use type diversity index: According to the diversity-stability theory[10], the land system stability indicates the land ecological security. Thus, the land use type diversity has a critical direct impact on cultivated land ecological security. The formula of the land use type diversity index can be obtained as follows:

$$N_{i}=-\sum_{k=1}^{n}L_{k}\text{ln}(L_{k})$$ (2)

Where *N*_*i*_ is the land use type diversity index of the *i*th area, *n* is the total land use type number, and *L*_*k*_ is the *k*th land use type area.

#### Data normalization

All indexes proposed in the index system must be standardized to overcome incompatibilities among the parameters since they are different in dimensions. In general, the normalization method and standard value method are used to eliminate the dimension differences. A combination of both methods is adopted in this study, in which the standard value method[36] is used if the indicators have standard values from international, national or Yunnan Province and the min-max normalization[22, 37] method is used when its standard value is absent. Because the index system is composed of two subsystems, and eco-pressure indicators and eco-support indicators are calculated separately, every index is a positive index in the corresponding system. Eq 3 is applied as:

$$Y_{ij}=\frac{Y_{ij}^{\prime }-Y_{j\text{min}}}{Y_{j\text{max}}-Y_{j\text{min}}}$$ (3)

Where *Y*_*ij*_ is normalized index value, $Y_{ij}^{\prime }$ is the original index value, *Y*_*j*max_ is the maximum value of the *j*th index, and *Y*_*j*min_ is the minimum value of the *j*th index.

#### Determination of index weight

The entropy method is increasingly employed in the weighting process of evaluation indicators since the weight can be objectively given to avoid the influence of human subjective factors[38]. Thus, the entropy method is applied to obtain the weight of the assessment index in this paper through MATLAB2016a. The formulas are as follows:

$$H_{j}=-\text{ln}{\left(n\right)}^{-1}\sum_{i=1}^{n}f_{ij}\text{ln}f_{ij}$$ (4)

$$f_{ij}=X_{ij}/\sum_{i=1}^{n}X_{ij}(j=1,\cdots m)$$ (5)

$$w_{\text{j}}=(1-H_{j})/(m-\sum_{j=1}^{m}H_{j})$$ (6)

Where *i* stands for the *i*th year, *j* stands for the assessment index, *X*_*ij*_ is the standardized criterion of the *j*th index in the *i*th year, *f*_*ij*_ stands for the proportion of the overall evaluation years, *H*_*j*_ stands for the entropy of the *j*th index, and *w*_*j*_ stands for the weight of the *j*th index. According to the formulas, the weight of all those indexes can be obtained (Table 1).

#### GA-BP neural network assessment model

**Fig 3:**
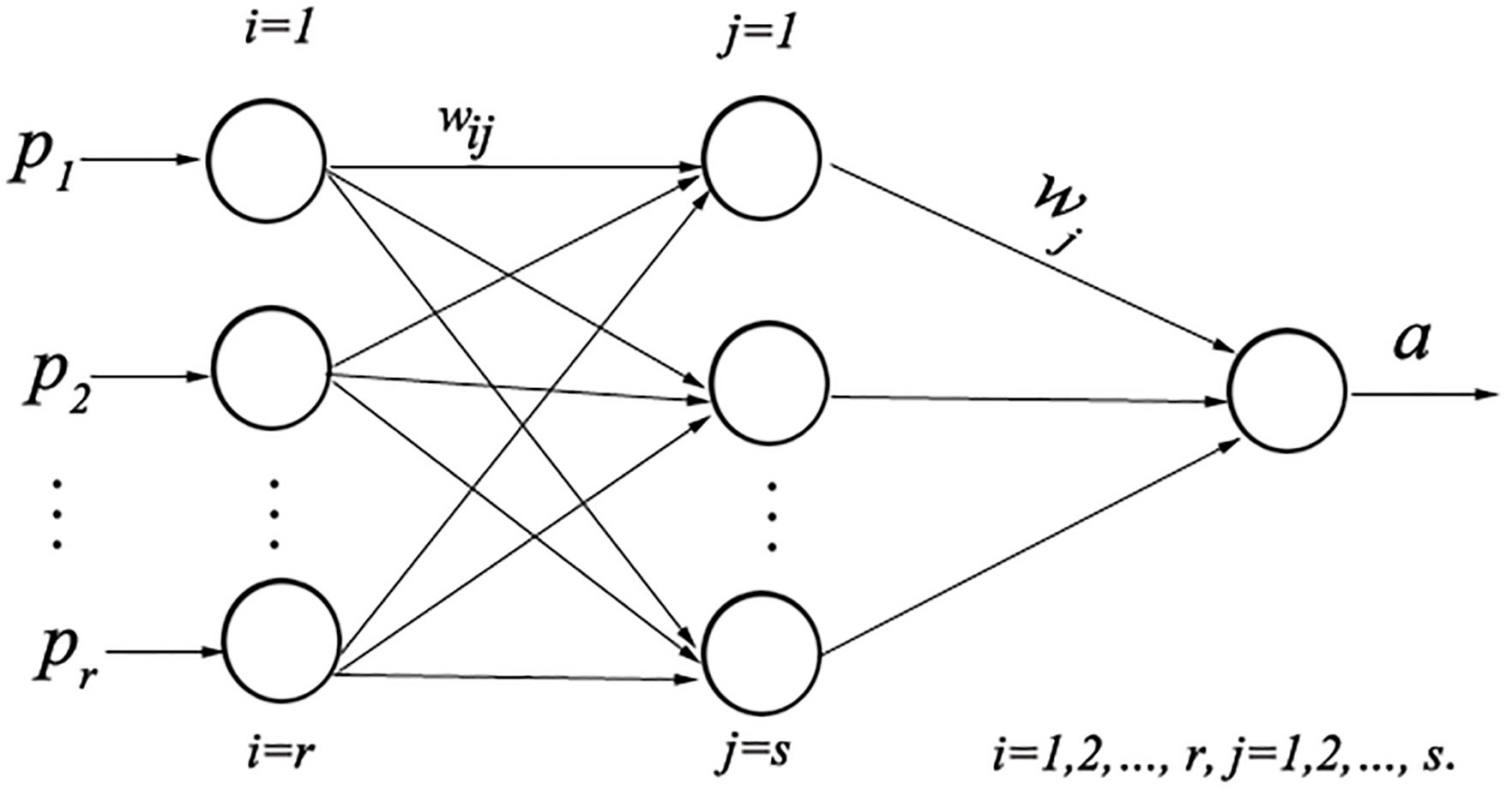
BP neural network structure. The BP neural network structure has 3 layers. *P*_*i*_ is the input data set, *p*_*j*_ is the hidden layer node, a is the actual output, *w*_*ij*_ and w_*j*_ are the weights.

A back-propagation artificial network (BP network) is a multiple layer, feed forward neural network[39], which consists of an input layer, hidden layer and output layer. The design of BP neural network includes input/output variable handling, network topology designing and network training parameters determination; its structure is shown in Fig 3. In this paper, a BP neural network is first used to assess and analyze the cultivated land ecological security. However, although a BP neural network with nonlinear mapping, self-learning and a self-adaptive ability is suitably applied to an evaluation of the cultivated land ecological security, the convergence rate of the algorithm is very slow and may have a local minimum problem. Thus, to avoid these defects, the genetic algorithm (GA) is introduced to improve the program model. Furthermore, a genetic algorithm is used to optimize the initial weights and the threshold of the BP neural network and to establish the cultivated land ecological model based on the GA-BP network. In fact, the genetic algorithm has the ability to optimize the BP neural network[40]. GA has been used for function optimization, automatic control, combinatorial optimization, robotics, and parameter optimizations of neural networks, structure design, image processing models, and other areas. Therefore, the improved BP neural network model via genetic algorithm is more feasible in its application to evaluate the cultivated land ecological security.

To solve the problems of arbitrary initial values in the BP neural network, a genetic algorithm is chosen to optimize the BP neural network. Now, the introduced GA-BP is as follows.

**Fig 4:**
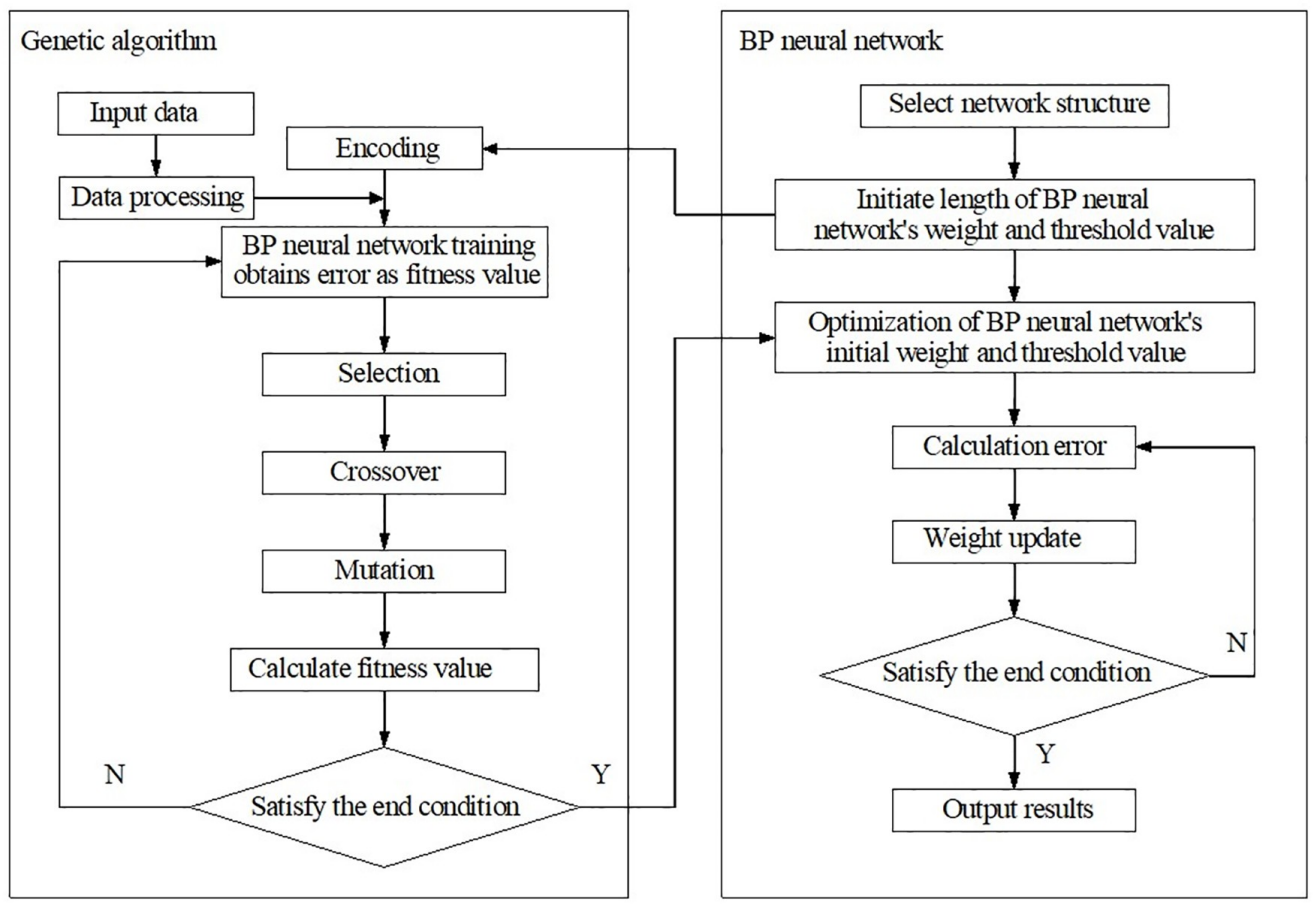
GA-BP neural network process. Optimizing weights and the threshold of the BP neural network by GA is divided into two steps. The first step used genetic algorithm embedding neural network and searched the best individual in the probable scope of the weights of BP network. The second step continued to apply BP neural network.

This section mainly describes the study method under the premise of a fixed BP network structure; that is; firstly, GA will be used to optimize initially connected weights and the threshold of the BP network, and then uses the BP algorithm to accurately search the optimal solution of the weight and the threshold. The flowchart of genetic algorithm is shown in Fig 4.

Step 1. Encoding, initial random fitness: using GA, each individual of the population must be described in a chromosome representation. Here, according to the actual situation in the article, the number of nodes in the input layer is *m* = 18, and it is *n* = 1 in the output layer. The BP network structure is 18-8-1. As a result, the multi hidden layer network structure is more complicated and the three neural network layers can implement almost all pattern recognition and classification tasks; the three-layer neural network is thus employed. Formula $h=\sqrt{m+n}+a,a\in [1,10]$ is widely used for the estimation of hidden layer nodes, and the results will be determined through a set of experiments.
Step 2. Fitness function: GA uses the fitness function to evaluate the viability of the chromosome. The chromosome length is *l* = *m* × *h* + *h* + *h* × *n* + *n*.
Step 3. Selection: the most important step in GA is the selection. In this paper, Roulette wheel selection is employed.
Step 4. Crossover and mutation: both crossover and mutation can create new individuals via recombining or mutation.
Step 5. The process will be accomplished when the appropriate fitness is obtained or the evolution has completed the default maximum number of generations. The output of this process is the individual with the best fitness, and this individual consists of the weights and threshold. The weights and threshold will be used as the initial setup to train the BP neural network.

The evaluation model of the GA-BP neural network is trained, learned and tested by applying the neural network toolbox of MATALAB2016a and writing the GA-BP source program. Conclusions showed that the evaluation system of the GA-BP neural network obtains a higher precision in a shorter period of time and it is obviously better than the BP neural network in the convergence rate, accuracy and stability draw by comparison.

#### Grading standards for ecological security

Based on the GA-BP neural network and combined entropy weight results, cultivated land EPI and ES’I of Yuxi City from 2005 to 2015 can be calculated. Then, the ESI can also be computed with the following formula:

$$ESI=\frac{ES\prime I}{EPI}$$ (7)

Where ESI stands for the ecological security index, ES’I stands for the ecological support index, and EPI stands for the ecological pressure index. When ESI = 1, the ecological support subsystem can barely balance the pressure subsystem, and make cultivated land kept at a critical safety level. If ESI>1, then the ecological support subsystem not only can balance the pressure system but can also supply more ecological services. However, when ESI<1, the cultivated land system is unsafe as too many ecological pressures throw off the system balance. According to these criteria, 5 degrees can be classified, and the classification standards and security grades are shown in Table 2.

**Table 2:** Grading standard for a cultivated land ecological security assessment in Yuxi City.

Grade of security	ESI	Characteristics of the cultivated land ecological system
**I** (highly safe)	>2	complete structure; strong function; high fertility; less pollution; higher forest cover
**II** (safe)	1–2	more complete structure; better function; higher fertility; low pollution; higher land use degree
**III** (critically safe)	0.8–1.0	deteriorating trend; basic function; emergence of ecological problems
**IV** (risky)	0.5–0.8	structural deterioration; insufficiency function; more ecological problems
**V** (very risky)	<0.5	incomplete structure; decreased function; severe ecological disaster

## Results

### Determination of flatland and mountain land area

Land area proportions are above 50% or approach 50% in Hongta, Jiangchuan, Tonghai, Chengjiang and Huaning counties, but they are below 40% in E’shan, Yimen, Xinping and Yuanjiang, where the slope varied from 0° to 15° (Table 3). The land area proportions where slope varied from 15° to 25° are all above 30% in the latter four counties, except E’shan County where the slope varied from 25° to 75°. Meanwhile, the topographic reliefs of the first five counties are from 0 to 20 m, and the land area proportions are all above 40%. However, the proportions of the latter four counties in the same topographic relief ranges are only 20%, and approximately 60% where the topographic relief ranges from 20 to 50 m. Based on these conditions, Yuxi City can be divided into two different geographic spaces that called flatland and mountain land counties according to the terrain characteristics of slope and topographic relief. Hongta District, Jiangchuan District, Tonghai County, Chengjiang County and Huaning County, which locate in the eastern Yuxi City, belong to flatland areas, but E’shan County, Yimen County, Xinping County and Yuanjiang County, which locate in the western Yuxi City, are mountainous areas.

**Table 3:** Land area proportions of different slope and topographic relief in counties.

Counties	Proportion of land area in different slopes			Proportion of land area in different relieves		
	0-15 (°)	15-25 (°)	25-75 (°)	0-20(m)	20-50(m)	50-300(m)
**Hongta**	52.20	28.68	19.12	43.03	48.42	8.55
**Jiangchuan**	64.11	24.43	11.46	69.87	27.20	2.94
**Tonghai**	55.38	27.25	17.36	48.96	43.79	7.25
**Chengjiang**	59.93	25.07	15.00	66.61	28.93	4.46
**Huaning**	48.15	34.88	16.97	55.51	37.51	6.98
**E’shan**	39.23	36.06	26.34	22.49	62.44	15.07
**Yimen**	32.42	34.00	33.58	24.95	58.24	16.81
**Xinping**	28.53	36.10	35.37	22.39	61.00	16.61
**Yuanjiang**	27.38	33.88	38.74	20.98	59.39	19.63

**Fig 5:**
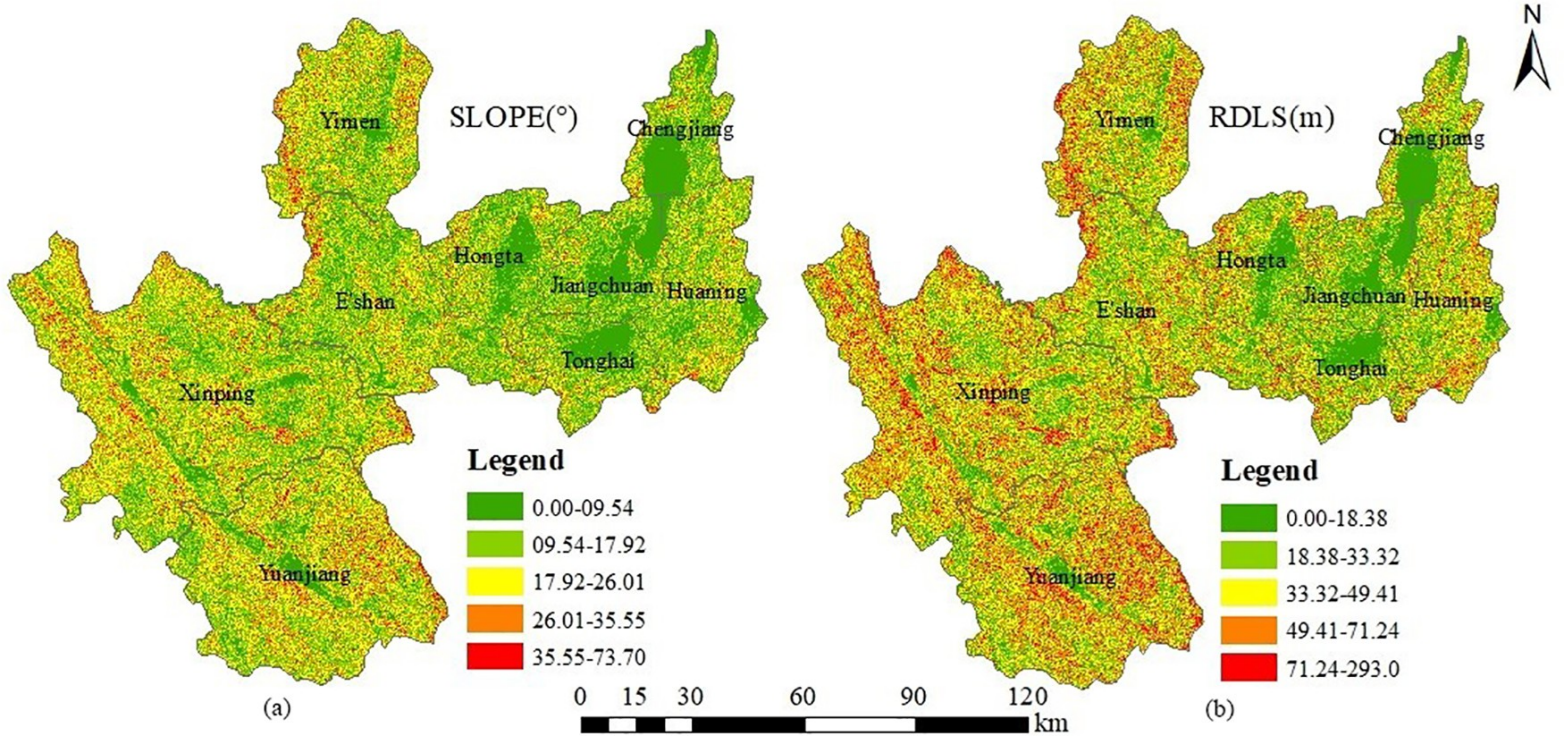
Spatial distributions of different slope and topographic relief in Yuxi city. Natural break point method is adopted in classification on slopes and reliefs by using ArcGIS 10.5.

Otherwise, the slope and topographic relief are classified into five grades to obtain the spatial distribution of different slope (Fig 5a) and topographic relief grades (Fig 5b) in Yuxi City via ArcGIS 10.5. The graph shows that a lower slope area corresponds to a lower topographic relief area and a higher slope area corresponds to a higher topographic relief area. When the two vector graphs are intersected, land areas above 60% at different slope grades are completely consistent with the same topographic relief grade, which verified the feasibility of classifying flatland counties and mountain land counties with the land area proportions of different slopes and topographic reliefs.

### Characteristic of land use/cover change

#### Temporal variations of land use/cover

**Fig 6:**
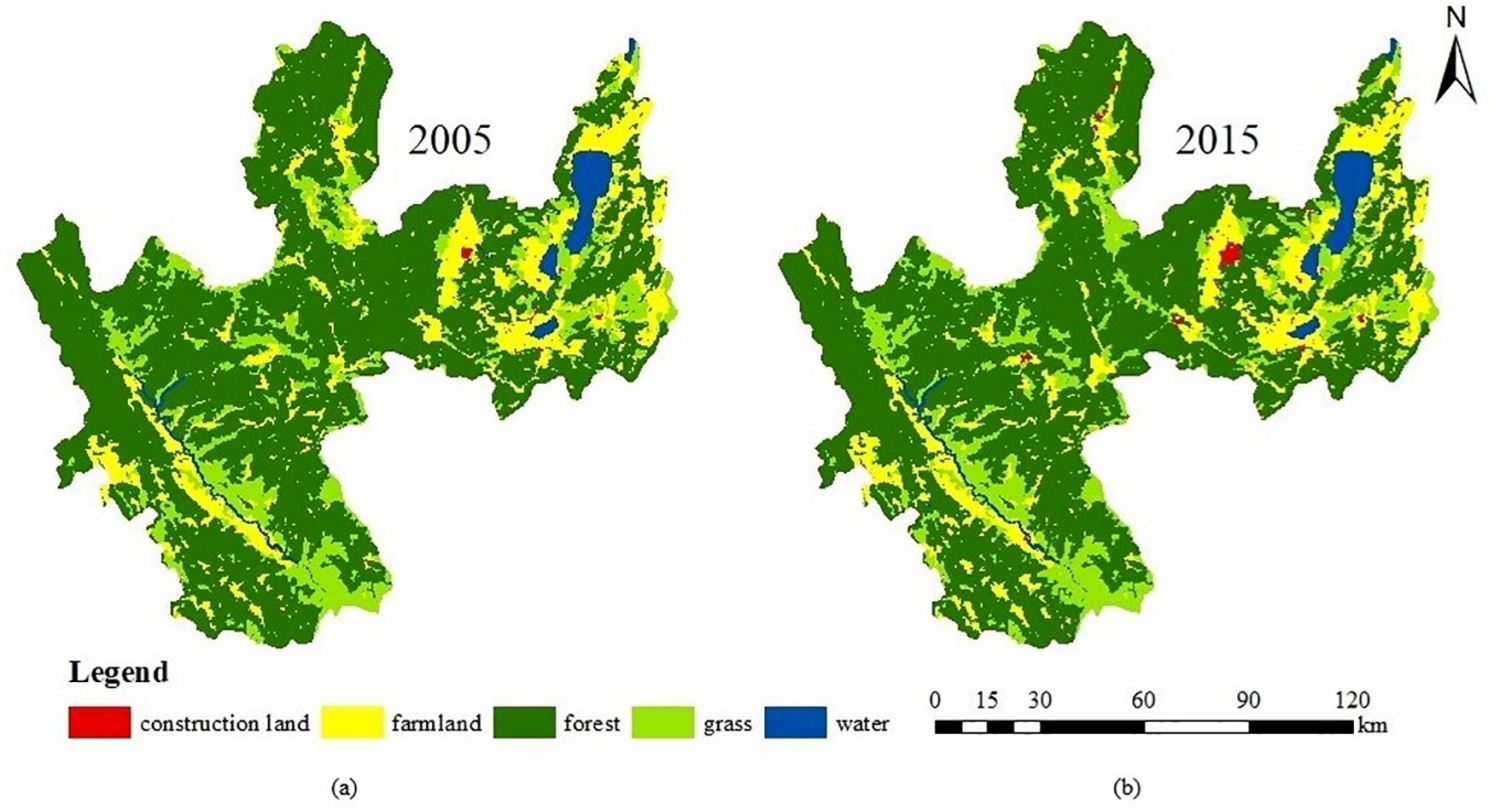
Land use/cover change from 2005 to 2015 in Yuxi City. The image of land use map is obtained by using ArcGIS 10.5.

With the aid of ArcGIS 10.5, the translated graphic map of 2005 interacted with the 2015 graphic map, which gave rise to the statistics in Table 4. As is shown in Fig 6 and Table 4, from 2005 (Fig 6a) to 2015 (Fig 6b), the forest type takes the top coverage for Yuxi City and accounts for more than 58% of all land. Notably, construction land area greatly increased by 6036.30 hm^2^ during the 10-year periods, with its percentage increasing from 1.15% to 1.56%. The single land use dynamic index of construction land use is 3.4902. The farmland change shows an obvious lowering tendency, with a rate from 20.28% in 2005 to 19.59% in 2015, and the land use dynamic index is -0.3375. The other land use types illustrate relatively little change, such as the grass land being slightly down, while the forest and water areas increase slightly.

**Table 4:** Land use/cover types of 2005 and 2015 in Yuxi City.

Land use/cover type		Farmland	Grass	Forest	Water	Construction land
**2005**	**Area (hm**^**2**^**)**	304005.83	270204.25	870343.65	37555.67	17295.10
	**Proportion (%)**	20.28	18.02	58.05	2.50	1.15
**2015**	**Area (hm**^**2**^**)**	293746.06	268666.17	876066.14	37594.73	23331.40
	**Proportion (%)**	19.59	17.92	58.43	2.51	1.56
**Single land use dynamic index**		-0.3375	-0.0569	0.0657	0.0104	3.4902

The increase in construction land use in Yuxi City during the 10-year period is mainly at the expense of cutting down the forest. Thus, by further analysis of Table 5, the changing rate of construction land adds up to the highest point by 33.69% from 2005 to 2015, and farmland contributes the most to the accumulation of construction land, accounting for 82.12%.

**Table 5:** Land use/cover conversion matrix from 2005 to 2015 in Yuxi City (hm^2^).

Land types	Farmland	Grassland	Forestland	Water	Construction land	Total (2005)
**Farmland**	268762.30	13108.63	16915.31	262.53	4957.06	304005.83
**Grassland**	6418.78	227968.08	34187.93	140.49	1488.96	270204.25
**Forestland**	17012.38	27293.90	824461.51	194.67	1381.19	870343.65
**Water**	142.38	167.40	256.77	36956.27	32.85	37555.67
**Construction land**	1410.21	128.16	244.62	40.77	15471.34	17295.10
**Total (2015)**	293746.06	268666.17	876066.14	37594.73	23331.40	1499404.50

According to calculate the data in Tables 4 and 5, the construction land conversion rate has increased to 10.54%, which can be attributed to the farmland rearrangement and reclamation, thereby adding a farmland area of 1410.21 hm^2^ and it comprises 77.32% of the total conversion area. Farmland changes the most after construction land, although the conversion indexes of grass in and out are both higher than those of farmland, reaching above 15%. Even if the conversion index of grass fluctuates greatly, its total changes slightly. However, the conversion rate in and out of farmland is 8.51% and 11.59%, respectively, and thus, the total acreage of farmland decreases during the 10-year periods as it is mainly transformed from the forest and grassland.

#### Spatial variations of land use/cover

**Fig 7:**
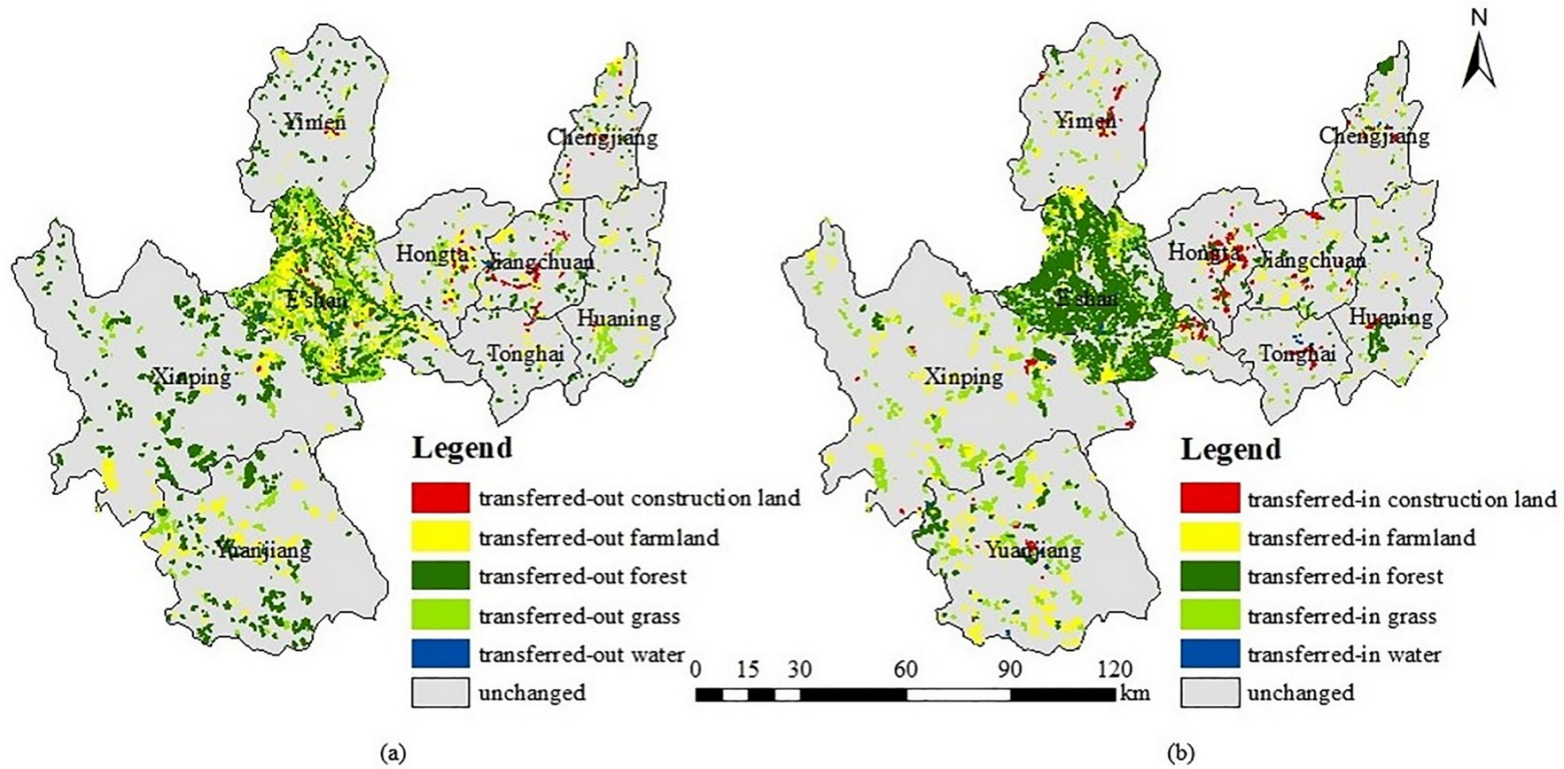
Spatial change in land use/cover in Yuxi City from 2005 to 2015. There are transferred-out areas in (a) and transferred-in areas in (b). Different colors refer to different change types.

Through interacting the 2005–2015 administrative maps with the computer graphic map of Yuxi City, a series of land change statistics of different types in different places is shown in Table 5 and Fig 7. The transferred-out data are shown in Fig 7a and transferred-in data are shown in Fig 7b. From the perspective of place, farmland, forest and grassland changes mainly in E’shan County, while the construction land use change focuses on the Hongta and Jiangchuan districts. Specially, the areas of grassland transferred-in and out both have the highest rates in E’shan County, accounting for 38.17% and 52.77% of farmland conversion, respectively. Yuanjiang County changes second-most in farmland transferred-in and out, with the rates of 27.32% and 16.14%, respectively. Hongta District has the highest rate of transferred-in construction land at 2552.94 hm^2^, accounting for 32.48% of all transferred-in construction land. Conversely, Jiangchuan District holds the most transferred-out construction land, with an area of 851.13 hm^2^, taking 46.67%. However, E’shan County still fluctuates the most in both construction land transferred-in and out with areas of 1188.36 hm^2^ and 300.69 hm^2^, respectively. Additionally, E’shan County has the most forest transferred-in area, accounting for 73.12% of the total transferred-in land of Yuxi City. Meanwhile, Xinping County has the most transferred-out forestland, accounting for 33.66% of the total transferred-out forest in Yuxi. Overall, the change of water is negligible, and Tonghai County transferred in the most area and E’shan County transferred out the most, with areas of 161.82 hm^2^ and 344.70 hm^2^, respectively. In conclusion, the land use in E’shan County fluctuates the most, with Jiangchuan District, Xinping County, Hongta Distrcit, and Yuanjiang County following in order, and Huaning County, Yimen County and Chengjiang County change the least. Additionally, for the western part of Yuxi City, including E’shan, Xinping, Yuanjiang and Yimen County, the main change of its land is in forest and grassland, while the eastern counties, such as those in Hongta District, Jiangchuan District, Tonghai County, Huaning County and Chengjiang County, changes are mostly in farmland and construction land.

As shown in Fig 7 and analyzed above, there are significant differences in LUCC between the east and west of Yuxi City, which are flatland areas and mountainous areas. In the flatland areas, the transferred-in and transferred-out areas mainly came from the construction land and farmland, but in the mountainous areas, the transferred-in and transferred-out areas mainly came from the forest and grassland. The main reason is that the east of Yuxi City is flat and has better hydrothermal conditions when compared with the west. Flatland areas are suitable for farming and construction and the social economy of these areas has been developed rapidly in recent years. Therefore, the differences in LUCC between flatland areas and mountainous areas could be from the differences of natural conditions and social economic development.

### Cultivated land ecological security change

#### Cultivated land ecological security changes in Yuxi City

According to the GA-BP model, the EPI and ES’I of the cultivated land from 2005 to 2015 in Yuxi City can be obtained (Table 6), then, with the help of formula (7), ESI for the study period can also be calculated. The bigger the EPI is, the more ecological pressures present, and the bigger ES’I is, the stronger support of the system presents. Thus, a bigger ESI means a more secure cultivated land ecological system.

**Table 6:** Change of cultivated land ecological security from 2005 to 2015 in Yuxi City.

Year	Ecological pressure index (EPI)	Ecological support index (ES’I)	Ecological security index (ESI)	Security grade
**2005**	0.3172	0.3305	1.0421	II
**2006**	0.3007	0.3061	1.0178	II
**2007**	0.2779	0.3323	1.1956	II
**2008**	0.2656	0.3201	1.2053	II
**2009**	0.3045	0.3089	1.0146	II
**2010**	0.3040	0.2969	0.9768	III
**2011**	0.2956	0.3146	1.0645	II
**2012**	0.2903	0.3030	1.0437	II
**2013**	0.2767	0.3036	1.0971	II
**2014**	0.2725	0.3015	1.1063	II
**2015**	0.2639	0.3032	1.1488	II

Table 6 shows that both the ecological pressure index and ecological support index are low, but it’s high in the ecological security index, and its security grade stays at level II, which indicates a relatively healthy ecological condition. During the study period, the ecological security index of farmland tends to increase and then decrease, and it obviously relates to the index of ecological pressure, thus displaying a notable negative correlation with a Pearson index of 0.829. The main reason for such a change is because the use of fertilizer of each unit of land increases and then decreases, and the rates of water loss and soil erosion have been controlled by adding forest and grassland from 2012 to 2015. During the 10 year-studied, the farmland area of Yuxi City mostly decreased, but the decreased farmland was converted into forest. Overall, the ecological condition of Yuxi City tends to be improved and the ecological pressure stays at a low level, which leads to the result that the ecological security of Yuxi City was always at a state of II during the past 10-year study periods.

#### Cultivated land ecological security changes in different counties

**Fig 8:**
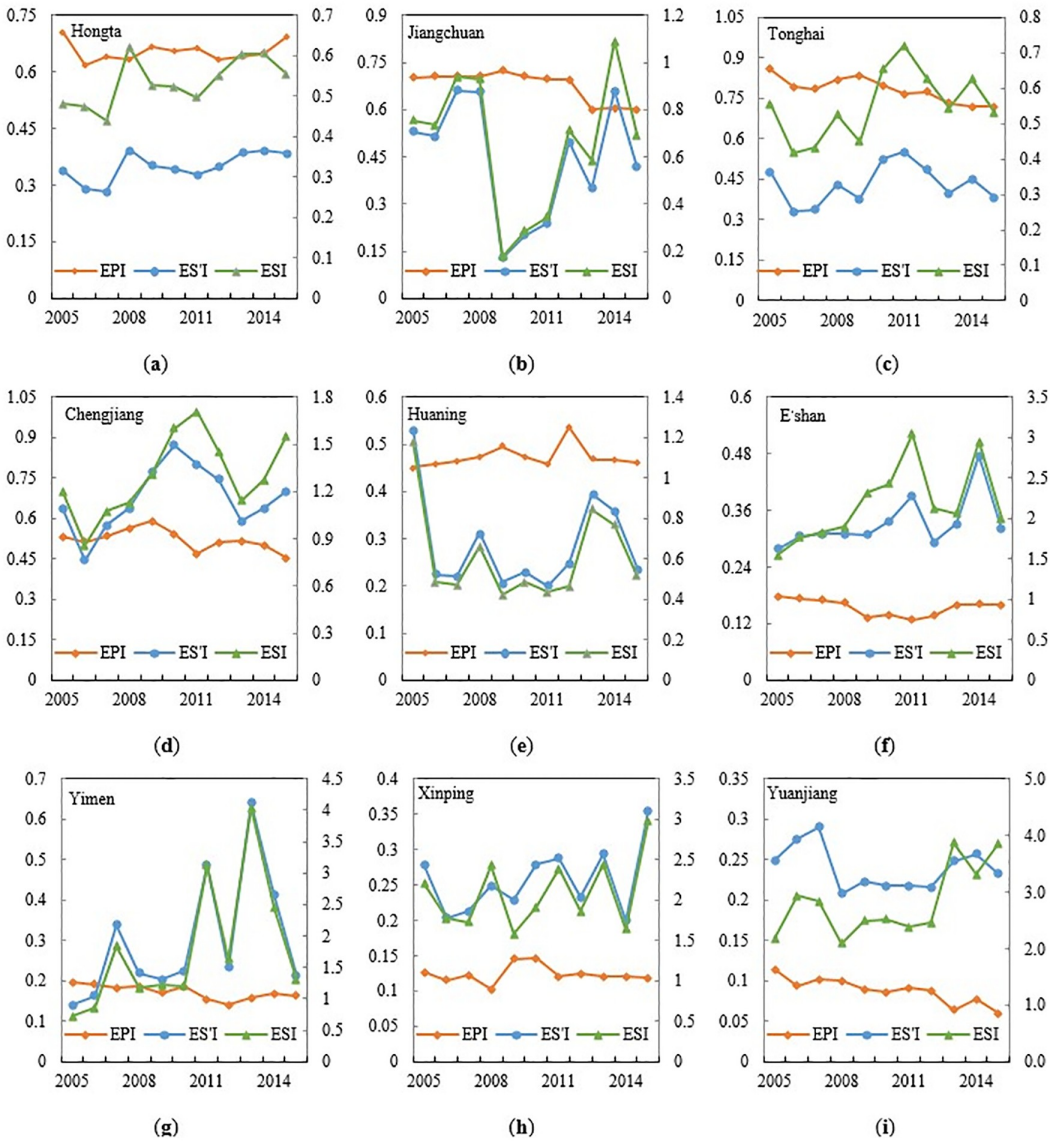
Variation trends of ecological security of different counties. Three broken lines refer to the changes of eco-pressure, eco-support and eco-security states of every county in Yuxi City respectively.

For all other counties and districts in Yuxi City, except Yuanjiang County, the ecological security index and ecological support index changed at the same pace (Fig 8). By further analyzing the Pearson correlation[41]. of the three indexes in SPSS 23.0, the ecological support index and ecological security index are notably positively (P<0.01) related in counties and districts except Yuanjiang County, while in E’shan County, the situation is slightly different by showing an obviously negative correlation (P<0.05). However, in Yuanjiang County, the farmland ecological security appears to be unrelated to its support security index, but shows a negative correlation to its ecological pressure index (P<0.01).

Hongta District is the economic and cultural center of Yuxi City, with the largest economic gross, which also means that it will bear the most ecological pressure. However, with the calling for building an ecologically friendly city, the ecological pressure index shows a steadily decreasing tendency while the ecological security index and ecological support index slightly increase. This dynamic has mainly contributed to the effective curtailing of water loss and soil erosion, which was to 18.97% from 23.96%. However, due to its low ecological support index from 0.2 to 0.5 (Fig 8a), taking the land use/cover change into consideration, Hongta District had the highest number of transferred-in construction land, and this was transferred in at the expense of more farmland and forest. Thus, its ecological condition was affected and caused a higher ecological pressure index trend, which finally led to a low ecological security index, reaching the IV state, and in the most impacted areas, in a state of V.

Jiangchuan District has been established in recent years and has developed very quickly over three years, with its ecological security index first decreasing then increasing (Fig 8b). Particularly from 2008 to 2010, the ecological security was in the V state, which was caused by high rates of land destroy, soil erosion (47.15%) and fewer environment protection effects. However, with for the implementation of protecting Fuxian Lake, Jiangchuan District attempted to recover the land by reducing constructions and farmland, as 851.13 hm^2^ of the construction land and 46.67% of the total was transferred out. The area of construction land decreased by 179.70 hm^2^ and for farmland by 160 hm^2^, and the grassland area increased 1003.84 hm^2^. Therefore, the rates of water loss and soil erosion notably decreased to 24.34% from 47.14%, and the state of ecology demonstrated a curvy rising trend, from level V to IV and even III.

Abundant water and sunshine enable Tonghai County to be an ideal place to plant vegetables; however, during the 10-year study period, the farmland decreased by 432.08 hm^2^ and the construction land increased by 346.50 hm^2^. The total production and value of the vegetables rose in these years, and thus the use of pesticides, fertilizers and plastics films also increased, reaching the highest use rates among all the counties of Yuxi City. The pesticide load per unit cultivated area has been more than 20 kg/hm^2^ in a decade, fertilizer load per unit cultivated area has been more than 300 kg/hm^2^ and more than 330kg/hm^2^ in most years. And mulching film load per unit cultivated area was on the increase, from 38.43 kg/hm^2^ to 82.23 kg/hm^2^ for ten years, and even reached 90 kg/hm^2^ in 2012 and 2013. Moreover, its forestland decreased by 409.37 hm^2^, which made its ecological pressure index the highest (Fig 8c). Although the index of its ecological security climbed slowly, the level of its security stayed between IV and V.

Chengjiang County has the most ecological land use and has invested considerable resources in protecting the environment. Thus, its forest area increased 527.04 hm^2^ during the study period, making its ecological support index the highest among all the other areas of Yuxi City (Fig 8d). However, because both its water and soil loss and fertilizer burden rates are high, its water and soil loss has been rising from 36.35% to 58.64% and fertilizer was always around 300 kg/hm^2^. So, the farmland pressure indexes stayed above 0.5 and its level stayed at II during the 10-year study period.

Huaning County is the largest base of Yuxi City for planting oranges. Its farmland area decreased 474.71 hm^2^ and grassland areas decreased1080.96 hm^2^, as they were mostly converted into orchards for oranges. Meanwhile, the use of pesticides and fertilizers increased annually, the pesticide load per unit cultivated area increased from 11.31 kg/hm^2^ to 29.34 kg/hm^2^ and fertilizer load per unit cultivated area went up from 302.7 kg/hm^2^ to 477 kg/hm^2^, and reached to the highest in Yuxi City. Moreover, its recultivation rate has always ranked first, and its ecological pressure index increased to 0.4 during the research years (Fig 8e). Additionally, due to both forest coverage index and ecology land use index being relatively low, the index of ecological support is also low and changed the most from 0.2 to 0.4. All these results make the county’s ecological security condition risky or critically safe, and most of its lands are in the IV state.

E’shan County, Yimen County, Xinping County and Yuanjiang County are typical western mountain areas of Yuxi City, and the main land use/cover is the interconversion between forest and grassland. E’shan County is the main area for reforestation, while Yuanjiang and Xinping County increased their farmland areas by more than 1000 hm^2^. The economic development levels of these places are relatively low, and both the ecological index and ecological pressure index for its farmlands are low, with a statistic lower than 0.2 (Fig 8f–8i). At the same time, because there are abundant forests and grasslands, the forest coverage rate and ecological land use rate are high, at more than 0.2, and thus the farmland safety level of the four counties are all at the II and I level, and the ecological condition is safe or highly safe.

## Conclusions

The land ecological security state is closely linked to LUCC, because land use and land cover changes largely alter terrestrial ecosystems and landscapes[42, 43], and bring further changes in climate, soil, vegetation and hydrology, which are the most important parts of the ecological environment. The variations of land use/cover change water and soil loss, productivity, consumption of chemical fertilizers via changing in the areas of various land types, local climate, soil fertility and so on, and to further alter cultivated land ecological security.

As two different kinds of geographic space, flatland areas and mountainous areas have different geographical conditions and lead to differentiated human activities. Then, differentiated human activities promote differentiated land use/cover changes and have a differentiated influence on local ecological security [32, 44]. Ecological pressures and ecological supports brought by different land use/cover changes in these two kinds of area generate relevant differences. Flatland areas are suitable for construction or farming because of their even terrain, so the areas of construction land or farmland accounted for a large proportion of their total land areas. Therefore, their cultivated land ecological pressure index values are high (0.6–1.0), but their support indexes are low (0.2–0.5) since the ecological environment is strongly influenced by human activities in these areas. Otherwise, LUCC shows a transformation between forest and grasslands in the mountainous areas. The ecological environments of these areas are barely affected by human impacts and their ecological security indexes are all higher than 1.

Of course, unlike human activities occurred partly because the natural conditions and partly because the government policy. Compared with mountainous areas, the flatland areas have relatively better physiography conditions and are easier to develop and construct into the center of human activities. At the same time, with the implementation of the ecological civilization construction policy, the mountainous areas should bear more responsibilities to balance the whole ecological system. Therefore, different geography spaces play different roles and take different responsibilities in social, economic and ecological systems [45]. According to the analyses on research results, it is clear that flatland areas have higher level of social and economic development but lower cultivated land ecological security, and the mountainous areas just the opposite. To guarantee the cultivated land ecological security in the whole area, different targeted measures should be adopted in the flatland areas and mountainous areas respectively. Because social and economic development is the foundation, flatland areas should pay its efforts to improve the living standards by increasing people’s income and more food substitutes to reduce pressures on cultivated land. In addition to these, intensive land use and greener production means should be taken in the flatland areas. And in the mountainous areas, the major measures are maintenance and protection. So, restoration of natural vegetation is the best choice in the mountainous areas.
